# P-2305. Study of Prescribing patterns and Effectiveness of Ceftolozane/Tazobactam [C/T] Real-world Analysis Results (SPECTRA): Results of the Immunocompromised Patients

**DOI:** 10.1093/ofid/ofae631.2458

**Published:** 2025-01-29

**Authors:** Emre Yucel, Alex Soriano, David Paterson, Florian Thalhammer, Stefan Kluge, Pierluigi Viale, Mike Allen, Brune Akrich, Yanbing Zhou, Huina Yang, Sundeep Kaul

**Affiliations:** Merck & Co., Inc., North Wales, Pennsylvania; Hospital Clínic de Barcelona, Barcelona, Catalonia, Spain; National University of Singapore, Singapore; Medizinische Universität Wien, Vienna, Wien, Austria; Department of Intensive Care, University Medical Center Hamburg-Eppendorf, Hamburg, Hamburg, Germany; Infectious Diseases Unit, Department of Medical and Surgical Sciences, Policlinico Sant'Orsola Malpighi, University of Bologna, Bologna, Italy, Bologna, Emilia-Romagna, Italy; MSD, UK, Ltd., London, England, United Kingdom; Merck Research Labs, MSD, Puteaux, Ile-de-France, France; Merck, Rahway, New Jersey; Tan Tock Seng Hospital, Singapore, Not Applicable, Singapore; Harefield hospital, london, England, United Kingdom

## Abstract

**Background:**

This sub-analysis of the SPECTRA (Study of Prescribing patterns and Effectiveness of Ceftolozane/Tazobactam [C/T] Real-world Analysis) study aimed to describe the clinical characteristics, treatment patterns, and outcomes of hospitalized adult immunocompromised patients.**Table 1.** Patient characteristics and Microbiology

**Figure:**
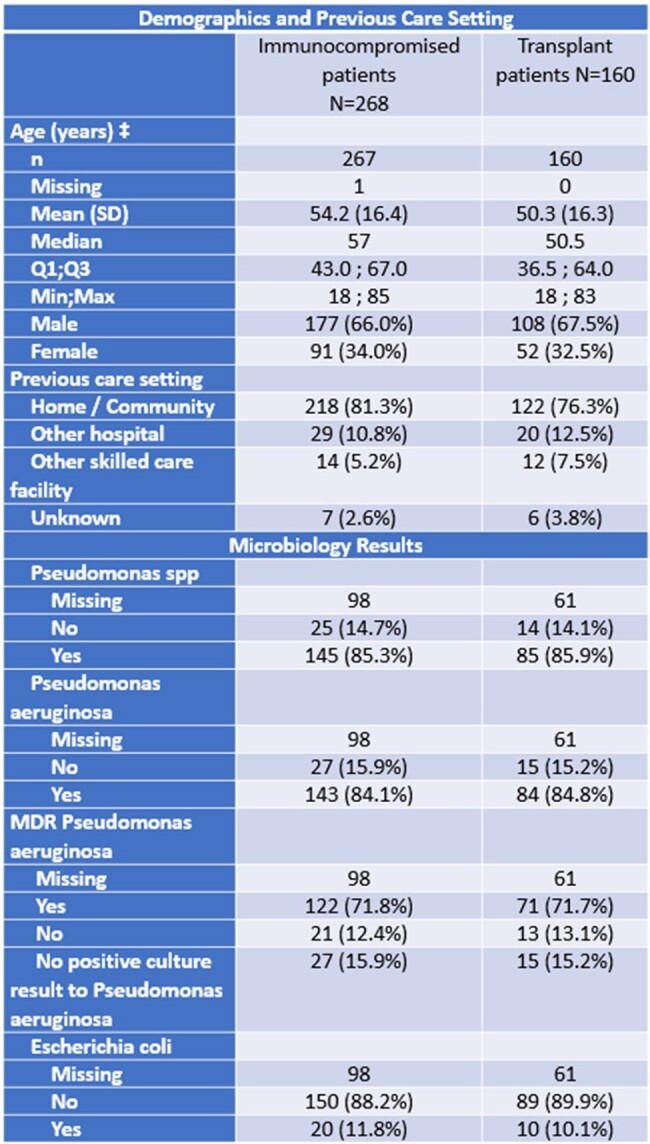
‡ For patients aged 90 or older the HCP was asked not to enter the exact age and to check a specific box. Therefore, patients aged 90 or older are included in 'Missing' and are not considered in the calculation of mean, median, etc. immunocompromised did not include chronic steroid use, age, or diabetes. Positive culture for more than one organism may apply for one patient.

Missing data correspond to patients with no microbiological (MB) sample for index infection, that is without any MB sample performed within +/- 14 days around the index infection with a positive culture result and at least one GN antibacterial tested (whatever the susceptibility result is).

**Methods:**

SPECTRA was a multicenter observational study conducted in 7 countries, with patients (age≥18yrs) who received ≥48-hours C/T treatment (n=617). Medical records were extracted covering a 6-month period prior to the index date, up to 30 days after the last dose of C/T or until death. All-cause in-hospital mortality (ACHM), clinical success, ICU admission, microbiology results, and treatment patterns in immunocompromised patients (IP, n=268) are reported. Immunocompromised patients were defined as patients with a Hematologic Malignancy or Solid Tumor or recipient of transplant. Transplant patients (TP) was 60% (n=160).**Table 2.** Clinical Outcomes: All-Cause In-Hospital Mortality and Clinical Success

**Figure:**
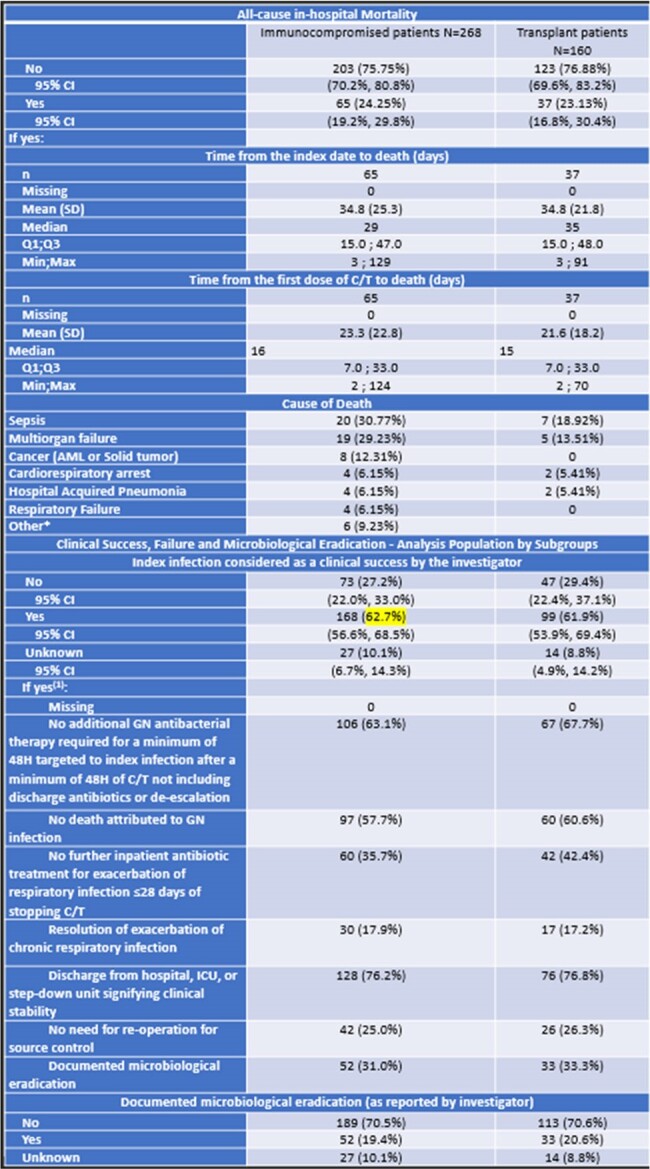
*Bile duct anostomic leakage, CMV infection, Post-operative Complication, Digestive hemorrhage, Invasive fungal infection, C difficile colitis (1) More than one criterion may apply for one patient.

**Results:**

Median age was 57 (IP) and 50.5 years (TP). 66% and 67.5% were male, respectively.28.4% had respiratory-related infections (RRI), 22.7% had sepsis, 27.6% had septic shock, 13.4% had Cystic Fibrosis, 29.4% had pneumonia, 2.6% had bone and joint infection. ACHM was 24% for IP, and 23% for TP. Time from index date to death was a median of 29 and 35, respectively. Leading causes of death were sepsis (31%), multiorgan failure (29%), cancer (12%) for IP. Clinical success was 62.7% for IP. 84% (143/170) were Pseudomonas aeruginosa (PsA), and MDR PsA was 71.8% (n=122). 38.7% (n=98) received optimal dose. Up to 50% of the patients were admitted to ICU during the index hospitalization (n=133), and 46% of the ICU admissions were related to index infection. Median length of stay at ICU was 13 days.

**Table 3.**
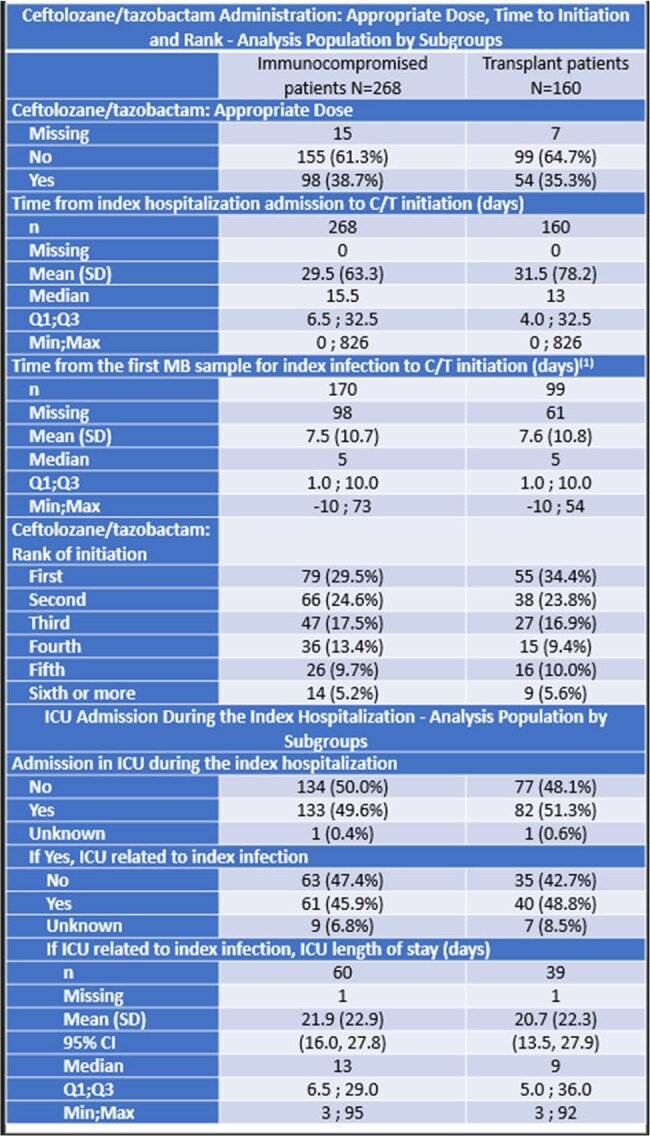
Treatment patterns and Intensive Care Unit (ICU)

**Conclusion:**

Treatment with C/T resulted in a high rate of clinical success in this real-world cohort of immunosuppressed patients, despite dosing that frequently deviated from labelled dosing. Pseudomonas aeruginosa including MDR isolates were frequently identified. Based on these results C/T is a useful option for the treatment of GN infections including those due to MDR PsA in the immunosuppressed patient population.

**Disclosures:**

Emre Yucel, PhD, Merck: I am a full time Merck Employee and own stocks in the retirement plan provided by Merck.|Merck: Stocks/Bonds (Public Company) David Paterson, bioMerieux: Grant/Research Support|bioMerieux: Honoraria|Merck: Advisor/Consultant|Merck: Grant/Research Support|Merck: Honoraria|Pfizer: Advisor/Consultant|Pfizer: Grant/Research Support|Pfizer: Honoraria|Shionogi: Grant/Research Support|Shionogi: Honoraria Florian Thalhammer, MD, MSD: Advisor/Consultant Stefan Kluge, Prof. Dr. med., Merck & Co: Advisor/Consultant|Merck & Co: Board Member Mike Allen, PhD, Merck: I am a full time Merck Employee and own stocks in the retirement plan provided by Merck.|Merck: Stocks/Bonds (Public Company) Brune Akrich, MD, Merck: I am a full time Merck Employee and own stocks in the retirement plan provided by Merck.|Merck: Stocks/Bonds (Public Company) Yanbing Zhou, PhD, Merck: I am a full time Merck Employee and own stocks in the retirement plan provided by Merck.|Merck: Stocks/Bonds (Public Company)

